# Paediatric oncology ward nurses’ experiences of patients’ deaths in China: A qualitative study

**DOI:** 10.1186/s12912-021-00720-1

**Published:** 2021-10-14

**Authors:** Ruo Han Ma, Xue Ping Zhao, Zhi Hong Ni, Xiao Ling Xue

**Affiliations:** 1 Children’s Hospital of Soochow University, No.92, Zhongnan St, Suzhou, 215025 China; 2 Medical College of Soochow University, No.199 Renai Rd, Suzhou, 215123 China; 3 School of Nursing, Medical College of Soochow University, No.1 Shizi St, Suzhou, 215000 China; 4 Global Institute of Software Technology, No.5, Qinshan Rd, Suzhou, 215163 China

**Keywords:** Children, Death, Cancer, Nurses, Paediatrics, Qualitative research

## Abstract

**Background:**

Considering cancer death is second only to accidental death in the number of lives claimed each year,nurses in paediatric oncology wards often experience helplessness, sadness, frustration and such other adverse emotions when they witness children’s death due to cancer.However,there is a lack of qualitative studies on nurses who witness the death of children in paediatric oncology wards in China.

**Method:**

A qualitative study was conducted using a semi-structured interview guide with 22 paediatric oncology ward nurses. Interviews were recorded and simultaneously translated and transcribed. Thematic analysis was used to analyse the data.

**Results:**

The analysis resulted in the identification of three main thematic categories: Different emotional expression, Different copingstrategies, A weak support system. Nursing managers should pay attention to problems faced by nurses in paediatric oncology wards, and take targeted measures in terms of continuing training courses, improving the psychological adaptability of oncology professional nurses, and providing them substantive support.

**Conclusion:**

Nurses in paediatric oncology wards have strong stress responses to facing the death of children. They reported experiencing complex psychological feelings and have different coping attitudes. Healthcare authorities should recognise and understand the needs of paediatric oncology ward nurses, who often witness the death of children. Appropriate and effective support measures should be planned and implemented for these nurses to maintain their mental health, thus enabling them to better serve patients.

## Introduction

The incidence of cancer in children is increasing and is the leading cause of death among children, second only to accidental death [1]. Globally, every year, more than 200,000 children are diagnosed with cancer before reaching the age of 15 years [2].

Globally, the incidence of cancer among children is 8.8/100,000, and the mortality rate is 4.3/100,000;in China, the figures are 6.9/100,000 and 4.4/100,000, respectively [3]. According to the Automated Childhood Cancer Information System and EUROCARE, leukaemia (34%), brain tumour (23%), and lymphoma (12%)are the largest diagnostic groups among children under 15 years old. The most frequent diagnoses are acute lymphoblastic leukaemia, astrocytoma,neuroblastoma, non-Hodgkin lymphoma, and nephroblastoma [1].

Cancer treatment includes surgery, radiotherapy, and chemotherapy. Children often require hospitalisation to undergo treatment. Children with cancer and their families have frequent contact with nurses during diagnosis and treatment,who establish an emotional connection with them. However,some children die during treatment [4]. Studies show that paediatric oncology nurses experience helplessness, sadness, frustration,and other adverse emotions when they witness children’s deaths [5, 6].

Attitude towards death refers to the relatively stable evaluative internal psychological tendency of an individual confronted with death [7]). As one of the main companions of dying patients, nurses play an important role in their end-of-life care. Their cognitions about life and attitudes towards death directly affect the quality of their work. Nurses in oncology departments are under various stressors, such as a tense working environment, instrument alarms, unstable patient conditions, and being responsible for patients’ lives. Oncology nurses’mental health is generally lower than that of nurses in general, and they face risks such as job burnout, post-traumatic stress disorder, and other mental health disorders [8].

Death is often viewed negatively, which can be emotionally draining [9, 10]. Oncology nurses may experience feelings of inadequacy, helplessness, defensiveness, or distress; and they employ varied coping mechanisms such as suppression and avoidance [11, 12]. This can potentially influence the quality of care nurses deliver and their job satisfaction, turnover, and attrition.

The Chinese cultural context is somewhat different from Western culture, exemplified by the Chinese saying,‘better a live coward than a dead hero’.However, death is believed to be a natural part of life, the Chinese avoid thinking or talking about death, as it may disrupt their inner harmony [13].

Up to 69% of oncology nurses in China experience depressive symptoms and 39%, anxiety symptoms [14]. Their psychological burden is significantly higher than the general population [15, 16].

Therefore, this study aimed to explore the cognitive, emotional, and behavioural aspects of paediatric oncology nurses’ experiences of patient deaths. This study could improve support for paediatric oncology nurses concerning children’s deaths. Furthermore, this study could improve understanding of nurses’ feelings and needs to provide them timely support to prevent negative emotions. Their experiences may assist healthcare providers in developing appropriate strategies to improve nurses’ expertise and ability to cope with patients’ deaths.

## Methods

### Ethical considerations

This study was conducted in accordance with the Declaration of Helsinki [17]. We confirm that all methods were performed in accordance with the relevant guidelines and regulations. Ethical approval was granted by the ethics committee of the Children’s Hospital of Soochow University, Suzhou City, Jiangsu Province, China (approval no. #2016045). Informed consent was obtained from all participants. Participants could decline answering specific questions and were free to ask for a break or terminate the interview at any point. The researchers consulted with professional psychologists. Only the interviewer knew participants’ identities, while the other researchers worked with anonymous data transcripts. All data were stored on a password-protected hard drive used only for this project. In addition, no one outside the research group had access to the data. Participants who wished to obtain the results were informed that they could do so later, but only as aggregated data.

### Design

The present study used a descriptive qualitative research design through semi-structured interviews [18]. The authors followed the Guidance on Qualitative Research Reporting Standards [19].

### Participants and setting

Interviews were conducted between January and March 2019 at paediatric oncology wards in three children’s hospitals in Jiangsu province, China. Purposive sampling was used to enrol paediatric oncology nurses who had experienced the death of child patients. Participants were initially recruited by recommendation of collaborating nursing managers. The inclusion criteria were as follows: (1) possession of a nurse practice qualification certificate, (2) more than 2 years of experience working in a paediatric oncology ward, (3) having a college degree in nursing, (4) experienced the death of a child patient with in the past year, and (5) provided informed consent to participate voluntarily.

We use nursing managers selection of participants due to confidentiality, voluntarily participation. The researchers had no pre-existing relationships with participants and were not involved in the care of Children with cancer at the hospital. All participants were provided with verbal and written information outlining the aims and methods of the study. The information statement reiterated that participation was voluntary and that all contributions would remain anonymous. Consent was obtained via a signed and dated written consent form which outlined that participants could consent to being interviewed, being audio-recorded, or both. Participants’ characteristics are presented in Table 1.

**Table 1:** Demographic data of the pediatric oncology nurses

No	Gender	Age	Professional years	Education
1	F	34	11	Junior college
2	F	36	13	Junior college
3	F	26	3	Junior college
4	M	29	16	Junior college
5	F	30	5	University
6	M	25	2	Junior college
7	F	28	5	Junior college
8	F	32	7	University
9	F	35	12	University
10	F	35	12	Junior college
11	M	30	6	University
12	F	39	19	University
13	F	35	12	Junior college
14	F	34	11	Junior college
15	F	33	8	University
16	F	32	5	University
17	M	29	6	Junior college
18	F	33	9	Junior college
19	F	27	2	University
20	F	29	5	University
21	F	30	3	University
22	F	28	5	Junior college

### Data collection

Participants were briefed about the study and informed of their right to withdraw at any point. Face-to-face interviews were conducted and audio-recorded in the participants’ private hospital offices at a time convenient to them. Written informed consent was obtained at the beginning of the interviews. A senior researcher (NZH) performed the interviews and trained less experienced co-workers. NZH is an experienced nurse whose highest credential is a PhD. All the researchers in this study were female and experienced in performing qualitative research.

A total of 22 interviews were conducted. The median duration was 50 min (32–106 min). An interview guide was developed and confirmed by the authors and was pilot-tested in the first three interviews, which resulted in minor revisions. These three interviews provided relevant information; therefore, they were included in the data analysis.

Each interview started with broad questions: (a) When was the first time you cared for a dying child? (b) What was the impact on you when you experienced the death of a child for the first time? (c) What problems did you encounter in the process until you could accept that the child had died? (d) How did you solve these problems? (e) What do you need the most help with after the death of a child?

All interviews were conducted in Chinese and were digitally recorded and transcribed by multiple research assistants fluent in Chinese. Recordings were transcribed verbatim within 24 h. Interviewing skills like active listening and open-ended questions were used. In addition, non-verbal information, such as obvious pauses, sobbing, and other speech features, were also recorded in the transcript.

### Data analysis

Twenty-two interviews were conducted and included in the analysis. In the last three interviews, no new data were generated, thereby achieving data saturation [20]. This study used thematic analysis—a data-driven method for identifying, analysing, and reporting patterns within data [21]. Braun and Clarke [21] stated that a thematic analysis can be used to find patterns in qualitative data and highlighted six phases: becoming familiar with the data, generating initial codes, searching for themes, reviewing themes, defining and naming themes, and producing the report. Audio recordings of the interviews were transcribed verbatim by the first author (MRH), and the third author (NZH) double-checked the transcripts. The first author (MRH), who is fluent in both Chinese and English, translated the interviews into English to make the data available to all of the authors. These interviews were read multiple times by all of the authors in order to become familiar with the data and gain deeper understanding of the content. In these interviews, all of the authors independently highlighted data extracts in line with the aim of the study and then discussed them to reach a consensus. Then the first author (MRH) identified the data extracts in line with the aim for the interviews and translated all of the data extracts into English. This translation was double-checked by the fourth author (XXL).

The authors then continued data analysis by independently coding the data extracts from all the interviews by writing notes and codes in the margins of the extracts. Several meetings were held among the authors to discuss and reach an agreement on coding. An initial thematic map was created based on the coding to form themes. Across the dataset, all authors found a sense of significance and relationships between the different themes. These themes were discussed, reviewed, and defined until an agreement was reached. Finally, three themes reflecting the content of the interviews were developed. Data analysis was conducted using NVIVO software (QST International, Cambridge, MA, USA).

### Methodological rigour

Credibility, dependability, confirmability, and transferability were considered to enhance the trustworthiness of this qualitative study [22]. Results were discussed with colleagues outside the research group (peer debriefing). To ensure dependability and confirmability, the interviewer had no previous contact with the interviewees. In addition, frequent discussions among the authors were held throughout the research processto enhance the dependability and strength of the results, especially those concerning the design phase, sampling, data collection, and data analysis. Quotations from the interviewees are presented in the results. We did our best to provide a detailed description of the context, participants, and data collection process to establish transferability. The transcripts were read and re-read until the researchers became familiar with the overall content. During this time, notes were made about potential codes. Data analysis involved the development of a list of codes that identified any feature of the data that was interesting and noteworthy. An inductive approach was adopted whereby coding was strongly linked to the data. Four researchers independently coded two transcripts, and a good level of inter-rater agreement was found. Three of the researchers then coded the remaining interviews, and fourth researcher was consulted where there were discrepancies. The codes were examined by all four researchers for ways in which they could be grouped to form themes or categories. In the final phase of the analysis, all researchers reviewed the data and agreed upon extracts that were representative examples of the themes that they had identified.

## Results

Through data analysis, we identified the following three themes: (1) different emotional expression, (2) different copingstrategies, (3) a weak support system (Table 2). Each theme is described below with supporting quotes from participants.

**Table 2:** Superordinate and sub-themes identified in the analysis

Themes	Sub-themes
(1) Strong stress response	Nervousness and worry
	Sadness and unwillingness to give up
	Loss and helplessness
(2) Different coping attitudes	Working hard and being conscientious
	Venting emotions
	Reflection and improving work quality
	Empathy
	Information avoidance
(3) Weak support system	Weak psychological support system
	Lack of knowledge

### Theme 1: different emotional expression

#### Nervousness and worry

When nurses deal with the death of children for the first time, they are often insufficiently prepared psychologically. They are often nervous and confused, they sweat and blush, and they present an elevated heart rate. Moreover, they are afraid of making a mistake when trying to resuscitate children.

*‘When the patient was dying, I was very nervous. My palms were sweaty and I was a little confused.’* –Nurse #16.

*‘During a patient’sresuscitation process, I was afraid that they had venous insufficiency, and that venepuncture might fail. I think I need to strengthen my venepuncture skill. When I gave the patient chest compressions, it really pulled at my heartstrings. I was afraid that the action was slow, or that I was doing it wrong. When the rescue was over, my hands were still shaking.’*–Nurse #17.

#### Sadness and unwillingness to give up

Owing to long-term interactions, relationships between nurses and children is deepened, and the latter’s deaths bring significant sadness to the former. Nurses often fail to accept that their patients have passed away; this is especially the case for nurses who are mothers.

*‘When the doctor declared that the child was dead, stopped resuscitation and removed the oxygen tube, my heart was torn. I have a son, and I know how it feels to be a parent. Seeing this felt very painful.’* –Nurse #18.

*‘I felt that a small life had just come into this world and disappeared too soon. I felt so sad, wishing he lived’.* –Nurse #5.

#### Loss and helplessness

The death of a child makes some nurses doubt their ability, resulting in feelings of loss and powerlessness. They put significant effort into caring for patients that could die at any given moment.

*‘I feltlost. Her mother had prepared a dumpling earlier, waiting for her to finish the lumbar puncture. But the child died and the dumpling remained uneaten.’* –Nurse #18.

*‘I could not help but look at the child. She kept alternating between consciousness and unconsciousness. I felt there was little I could do for the child. It was a very uncomfortable feeling, and it has been like this for a long time.’* –Nurse #19.

### Theme 2: different coping strategies

#### Working hard and being conscientious

Interviewees reported that they felt regret overtheir patients’ deathsunless they put all their effort into their work and treated every child with their utmost attention.

*‘The most important thing is work—to go all the wayand make no mistakes. We should have a clear conscience when dealing with children, especially when dealing with death and comforting bereaved family members. We should really do it with all of our heart. If we have a clear conscience, we will not be too sad later.’* –Nurse #20.

*‘A few days ago, a child was dying. I stayed with him all the time, pattedhis back, and made him feel better. There were no complications when I was on duty. It was psychologically comforting to me.’* –Nurse #21.

#### Venting emotions

Paediatric oncology nurses suppress their emotions in the process of dealing with children’s deaths, and only vent their negative emotions after the process is concluded.

*‘When I got back to the nurse station, I could not help it. My tears began to flow. After crying, I felt better.’* –Nurse #15.

*‘Sometimes, I thought about what the child said to mebefore dying. When I was at home, I couldnot avoid feeling sad and a little emotional. After crying, I slowly recovered.’* –Nurse #3.

#### Reflection and improving work quality

Some nurses repeatedly recalled the death of the child and reflected ontheir own shortcomings to improve their work.

*‘Every time I went home, I examinedmy work. For example, the child died of dyspnoea; was there some flaw in the resuscitationprocedure?’*–Nurse #9.

*‘Why did I not realise it until the end? If I had observed the child morecarefully, I might have been able to save the child.’* –Nurse #4.

#### Empathy

In the paediatric oncology ward, children’s smiles are one of the ways for nurses to obtain comfort. Nurses can effectively alleviate their negative emotions by focusing on happy children.

*‘The children are lovely. They cry only when they are sad. They are mostly very happy. I feel very happy when I see them smiling.’* –Nurse #1.

*‘I paid special attention to a child who looked like or had a personality similar to a patient I recentlylost.’*–Nurse #7.

*‘After the death of a child with cancer, I cherish the time I spend with my son, spend more time with him, talk to him, and I am patient with him. It feels good to be a part of his journey to become a grownup.’* –Nurse #12.

#### Information avoidance

Some nurses avoided confronting the death of their patients. They adopted negative coping strategiesand reduced the generation of negative emotions through avoidance.

*‘After the child died, I was in a bad mood. Sometimes I restrained myself from thinkingabout it.’* –Nurse #10.

*‘I did not like to talk to others. I ama very emotional person. When I talked about children’s death, I would cry easily. Sometimes I read the newspaper, and I dared not read the contents on children’s death.’* –Nurse #22.

### Theme 3: a weak support system

#### Weak psychological support system

The nurses faced great psychological stress when coping with patients’ deaths and were eager for understanding and support. After witnessing a death, nurses felt physical and mental exhaustion, and felt the need for emotional support*.*

*‘Sometimes I told my family about the death of children in the ward; they said I was pessimistic and I didnot likehearing that.’* –Nurse #6.

*‘It is really hard for me. I think I need to be comforted by other people. I need a hug; I want to hold someone for a while and cry.’* –Nurse #8.

Family members and friends do not understand the sadness of paediatric oncology nurses. Nurses do notreceive psychological support from friends and family members.

*‘Ordinarily, we comfort children and their families to be more open-minded and positive. However, when I am depressed, no one comforts me. I am forced to let feelings fade away with time.’* –Nurse #14.

#### Lack of knowledge

Although nurses hadrich clinical practice experience,in clinical practice, they relied solely on past experience in the hospice care of children and psychological care of their families, they lacked professional training, and faced many work-related challenges.

*‘I am not trained in psychological counselling to support the families of dying children. Sometimes I donot know how to tell their parents the truth, how to comfort them, and I feel that my job is redundant.’* –Nurse#11.

*‘I donot have a child of my own yet. To be honest, I cannot understand how parents feel when their children die. I donot know how to comfort them.’* –Nurse #4.

Nurses focused on the grief of parents after the death of children, rather than on their own grief and lack of coping knowledge.

*‘I think after the death of the child, their relatives are the most miserable. I feel sad for a few days but itis not a big problem. As time goes by, it gets better.’* –Nurse #2.

*‘After the child died, I couldnot be alone for some time. Whenever I was alone in aroom, I couldnot avoid thinking of him. For a long time, I couldnot come to terms with it.’* – Nurse #13.

## Discussion

The aim of the study was to explore the paediatric oncology ward nurses’ experiences of patients’ deaths in china. The results showed that paediatric oncology nurses developed strong emotional responses and profound grief after the death of a patient, which is consistent with prior results [23, 24]. Conte TM [24] found in her literature overview that nurses and children established an emotional connection during hospitalisation. After a child’s death, the lack of this connection causes grief. The source of this grief may be that these nurses are in a state of significant emotional stress and they strongly hope their patients will survive. If this fails,nurses can feel frustrated. They may even feel powerless or guilty because they could not save the child. Adwan JZ’s [25] results are in line with the results of the present study, which show that Nurses’ grief may be associated with increased job burnout, decreased job satisfaction,and increased turnover. Furthermore, support provided by nursing managers has a significant impact on job burnout in nurses who face patients’ deaths regularly.

Negative emotions are not conducive to nurses’ professional progress and personal development [26].Maslow’s theory of motivation emphasises that only when nurses are in a safe and protected environment can they fully develop professionally [27].

Thus, nursing managers should consider nurses’ professional and social roles and strengthen the emotional management provided to them. Specifically, nurses should be provided with psychological support. For example, the Los Angeles Children’s Hospital has established a grief support group [26], which holds bimonthly group activities and provides nurses with a safe space to share their experiences. Additionally, activities like yoga, massages, and going for a walk can help nurses’ grief management [28]. Other effective measures include a virtual network for nurses to share their sadness and obtain support [29], and imparting professional grief education to all paediatric oncology nurses [30]. Thus, nursing managers should understand the needs of nurses and arrange for death and grief education programs. The aim of continuing education should be to create a safe environment for nurses to talk regularly and form a grief support group, which includes psychological counselors, to provide support to nurses.

The results indicate that the coping styles of nurses differed, and their overall coping ability required improvement. After a child died, nurses adopted varied strategies, such as focusing on work, sharing their feelings with their colleagues, venting emotions, reflecting on improving the quality of care they provide, and shifting their attention to other things.

These results are similar to those found in a study concerning the coping strategies employed after children’s death due to cancer [4]. However, nurses’ coping strategies in many countries involve social support; religious support; and distraction through sports, hobbies, and entertainment; there are no negative avoidance strategies. Yang H found in her literature overview that these strategies may be related to differences in culture and personality, imperfect social support systems, and inadequate coping abilities [31].

Continued education for nurses often addresses patients’ deaths. However, distinct research concerning nurses in children’s oncology departments is lacking [32]. Therefore, we suggest that we should learn from other countries’ experience and design grief management measures adapted to Chinese culture to promote paediatric oncology nurses’ psychological health [33]. In addition, nurses should employ more effective and positive ways to cope with stress, improve their ability of psychological adaptation, and establish a positive occupational outlook. However, they should not be too hard on themselves, reduce their work pressure, choose their own leisure and entertainment methods, and adjust their mood.

The current results revealed that paediatric oncology nurses’ training lacks the necessary resource support, and training should address their emotionality and coping abilities. This is consistent with findings from Mohammed S et al. [34]. Nursing human resources are scarce, and the workload of nurses in paediatric oncology wards is heavy, thus, they are prone to burnout. It is suggested that the science of paediatric oncology be popularised, and the public’s awareness of paediatric oncology diseases be improved. More channels of information and education on cancer and its diagnosis, life, and death are required [35]. Nursing managers should focus on the evaluation of the workload of nurses in paediatric oncology wards [36]. Concerning self-control, there should be regular psychological consultations [37], opportunities to talk about negative emotions, and specialised training for paediatric oncology nurses,to provide them with efficient knowledge, coping strategies, and psychological counselling methods [38].

### Limitations

This study aimed to explore the cognitive, emotional, and behavioural aspects of nurses’ experiences of patient deaths. It is important to acknowledge certain limitations to the conclusions that can be drawn from this study. First, the sample is drawned in 3 hospital and not representative of the population of paediatric oncology nurses. Participants were initially recruited by recommendation of collaborating nursing managers. The recruitment procedures used in the study allow for the possibility of selection bias in the data collected.

Participants were included using convenience sampling. Participants were all currently employed nurses who had been undertaking professional development through university education. Nurses who were not enrolled in postgraduate education, those who had left nursing, and those not inclined to volunteer for interviews were not represented. Furthermore, the interview sample was made up of nurses aware that they would need to articulate their earliest memorable experience with patient death. Logistical restraints, including time, may have precluded a more in-depth analysis and integrated presentation of the large amount of data collected for this study.

### Clinical implications

Owing to rich emotional connections with their young patients, paediatric oncology nurses often have greater acute stress responses than other nurses, which may cause them to think about death more often. When nurses cannot cope with death, it affects the quality of their work, and leaves them unable to meet their patients´ physiological and psychological needs.

## Conclusion

Nurses in paediatric oncology wards feel stress after a patient’s death and they employ diverse coping mechanisms. Nurses employed both positive and negative coping strategies but were eager to obtain support. Nursing managers should apply strategies adapted to Chinese culture, include death and grief education in continuing training courses, and cultivate paediatric oncology professional nursing talents to master the required professional expertise.

## Data Availability

The data that support the findings of this study are available from the corresponding author upon reasonable request.
